# Mechanisms of NO_2_ Detection in Hybrid Structures Containing Reduced Graphene Oxide: A Review

**DOI:** 10.3390/s22145316

**Published:** 2022-07-15

**Authors:** Sabina Drewniak, Łukasz Drewniak, Tadeusz Pustelny

**Affiliations:** Department of Optoelectronics, Faculty of Electrical Engineering, Silesian University of Technology, 44-100 Gliwice, Poland; lukasz.drewniak@polsl.pl (Ł.D.); tadeusz.pustelny@polsl.pl (T.P.)

**Keywords:** rGO, reduced graphene oxide, NO_2_, SnO_2_, SnS_2_, ZnO, NiO, Fe_2_O_3_, CuO_2_, Polypyrrole, sensing mechanism

## Abstract

The sensitive detection of harmful gases, in particular nitrogen dioxide, is very important for our health and environment protection. Therefore, many papers on sensor materials used for NO_2_ detection have been published in recent years. Materials based on graphene and reduced graphene oxide deserve special attention, as they exhibit excellent sensor properties compared to the other materials. In this paper, we present the most recent advances in rGO hybrid materials developed for NO_2_ detection. We discuss their properties and, in particular, the mechanism of their interaction with NO_2_. We also present current problems occuring in this field.

## 1. Introduction—Problems of NO_2_ Detection

Dynamic urbanization, industrialization and development of agriculture vitally increases pollution of the natural environment. The polluted environment has a negative influence on human health. It causes many medical problems associated with consumption of contaminated food and water and breathing polluted air [1]. Therefore, the control of selected water and air parameters as well as adherence of actual standards set by the health organizations are crucial issues. One of the basic environmental threats is emission of nitrogen dioxide (NO_2_). NO_2_ is a highly reactive and toxic gas [2,3,4]. It is generated through combustion of fossil fuels (heating, power generation, engines in vehicles, etc.) [4], hence, is a serious challenge for the energy and automotive industries [3,5]. At concentrations currently measured in cities of Europe and North America, it causes an increase in symptoms of bronchitis in asthmatic children and reduces lung function growth [6,7], while at concentrations of 150 ppm it could even cause death [7]. Due to these threats, safety standards for NO_2_ exposures were formulated by many countries and world health organizations. For example, the United States Environmental Protection Agency established the annual exposure limit at 53 ppb [3,8], while the World Health Organization and European Union set it at even 21 ppb [3]. The need for monitoring low concentrations of NO_2_ in air made the development of NO_2_ sensors a leading topic of research.

For NO_2_ detection, various materials have been proposed as sensor layers, e.g., ZnO, WO_3_, SnO_2_, Fe_2_O_3_, etc. [9,10,11,12,13,14]. Generally, they exhibit excellent performance in NO_2_ detection, however, this is mainly at high temperature. High working temperature increases the energy consumption of the sensor, increases a safety hazard, degrades device stability and reduces the operating life. Therefore, there are attempts to replace them by other materials, free from these disadvantages. Realization of this goal is currently the hotspot of research.

## 2. Graphene and Graphene Oxide as a Gas Sensing Structure

**Figure 1:**
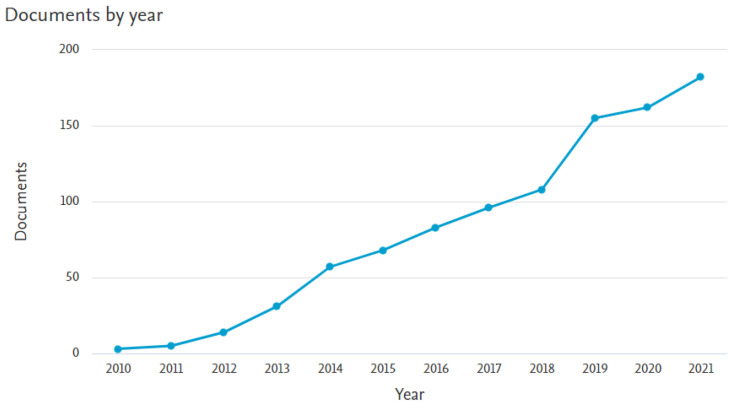
Number of articles per year devoted to the study of sensor properties of graphene oxide and reduced graphene oxide in aspect of sensor applications, prepared based on data from the Scopus database.

In recent years, special attention has been paid to graphene and graphene oxide [15,16,17,18,19]. An ideal graphene is a one atom thick layer arranged in a two-dimensional honeycomb lattice, composed of carbon atoms with sp^2^ hybridization [20,21,22]. The two-dimensional structure of graphene causes that it has a zero band gap and acts as a semimetal. It exhibits excellent mechanical, electrical, optical and thermal properties. Due to the huge surface area (~2630 m^2^g^−1^), high thermal conductivity (above 3000 W m K^−1^), high electron mobility at room temperature (~2 × 10^5^ cm^2^/V·s), carrier density (10^12^/cm^2^) and small resistivity (10^−6^ Ω·cm), it seems to be an ideal candidate for the sensor’s layer [20,22]. However, perfect graphene does not have dangling bonds, defects and additional functional groups, which have a strong influence on the gas adsorption [20,23,24]. Moreover, high production cost and low capacity of production methods make the application of pristine graphene still limited [25]. In this respect, derivatives of graphene [20,26], especially graphene oxide and reduced graphene oxides (rGO), are much more attractive. Therefore, the interest in these materials is increasing every year (Figure 1).

However, for sensor applications, rGO is more preferable because of higher conductivity and richer sorption sites compared to graphene oxide.

Reduced graphene oxide is a p-type semiconductor which consist of carbon, oxygen and hydrogen atoms [27,28]. The most common ratio of carbon to oxygen (C/O) is 12:1, although there are also reports where the value of 246:1 is given [29]. Oxygen and hydrogen are attached to the structure in the form of functional groups such as carbonyl (C=O), carboxyl (C-OOH), hydroxyl (C-OH) or epoxy (C-O-C) [8,30,31]. The number of functional groups is determined by (among others) a reduction method [29,32]. The reduction method can affect the response of the sensing structure due to the appearance of additional adsorption sites for gas [2]. For example, rGO fabricated using L-ascorbic acid has a higher degree of oxidation, a smaller amount of functional groups and a higher number of defects than rGO fabricated using hydrazine hydrate [2]. Therefore, the second oxide exhibits higher sensitivity for NO_2_ detection. Similar research on the influence of the reduction method and graphite precursor on the structure and physicochemical properties of rGO (in terms of the detection of selected gases, including NO_2_) were also carried out by the authors of this paper and published in [33,34].

**Figure 2:**
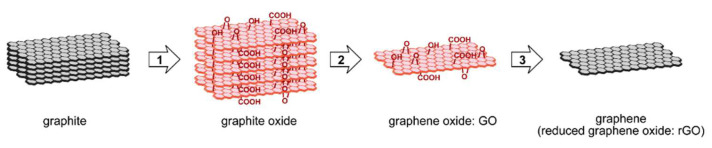
Scheme of fabrication process of reduced graphene oxide; (1) oxidation, (2) exfoliation, (3) reduction. Reprinted from Ref. [35].

The preparation of reduced graphene oxide is realized in three main steps: graphite oxidation, exfoliation of graphite oxide and reduction of graphite oxide sheets (Figure 2).

In the first step, the graphite precursor is oxidized to the form of graphite oxide [36]. Different methods are used for this purpose. The most known are Hummers’, Staudenmaier’s, Brodie and Tours’ methods [36,37,38,39,40]. During this process, additional oxygen containing functional groups (e.g., epoxy (C-O-C), hydroxyl (OH), carboxyl (R-COOH) and carbonyl (C=O)) are introduced, both to the edges of sheets and to the basal plane [36]. These groups increase the distance between the layers and the hydrophilicity of the layers [29]. In this case, an amount of carbon is from two to four times greater than the oxygen, depending on the preparation method [29]. The structure of graphite oxide consists of carbon atoms with sp^2^ and sp^3^ hybridization [29]. The carbon atoms are disordered due to attaching to functional groups [29], however, the graphene-like honeycomb lattice is preserved [29]. Obtained graphite oxide is an electrical insulator and is soluble in many solvents, both aqueous and organic [29]. In the next step, graphite oxide is exfoliated into monolayers or few-layered stacks of graphene oxide. This can be achieved by various thermal and mechanical methods [41]. The most common methods are sonicating or stirring in water. The aim of this process is to receive a monolayer (or at most several layers), which is called graphene oxide (GO) [42]. Finally, reduction is carried out. This is one of the most important processes, because it has a significant influence on the quality of produced material [29,40,43]. It determines how the structure of produced material is similar to pristine graphene. The reduction process removes functional groups [29,44] and defects in atomic-scale lattice [29], and recovers the conjugated network of graphitic lattice, as well [29]. The reduction process could be performed simultaneously with the exfoliation process [41] or as a separate process, e.g., using reductants, such as sodium borohydride, hydrazine, formaldehyde, sodium hydroxide and L-ascorbic acid [42]. The final product is called reduced graphene oxide (rGO) [37] or chemically modified graphene [41] (Figure 2). There are many methods to reducing graphene oxide. They can be classified into three main categories: chemical reduction, thermal reduction and electrochemical reduction. Depending on the method used, the obtained material may differ in properties. The main parameters used for estimating the effect of reduction are C/O ratio, structural defects and electrical conductivity [45]. Generally, chemical reduction produced rGO with a low surface area and low conductivity compared to the GO precursor. Thermal reduction produced rGO with a high surface area (close to that of pristine graphene) and with many structural imperfections (vacancies, voids and mechanical stress). Electrochemical reduction produced rGO the most similar to pristine graphene in terms of structure. The choice of the reduction method depends on the planned use of the rGO. In sensor applications, the electrochemical reduction is the most often used method, despite its toxicity. However, in our own research, we use the thermal reduction instead of chemical reduction because it is environmentally friendly and the reduction is performed simultaneously with exfoliation processes. Moreover, the degree of reduction process can be easily controlled by temperature [32].

**Figure 3:**
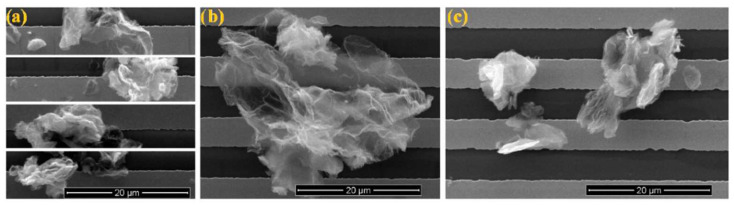
SEM images of reduced graphene oxide. (**a**) modified Hummers’ method and modified Tour’s method: (**b**) cloud-like particles and (**c**) compact particles. Reprinted from Ref. [34].

SEM images of graphite, graphene oxide and reduced graphene oxide are shown in Figure 3.

Despite the fact that reduced graphene oxide has an extremely large active area [46] and richer sorption sites than graphene and graphene oxide [47], it still has unsatisfactory gas sensitivity [46,48] and selectivity [8]. Therefore, to improve its properties, there are attempts to combine it with other materials used as receptor’s layers, e.g., semiconductors [8,49], semiconductor metal oxides (SnO_2_, ZnO, NiO) [46], conductive polymers [50], etc. In such structures, rGO is a doping material while the other components are the base materials. Usually, rGO concentration does not exceed a few percent. It is interesting that the literature uses various names for these types of materials, which can be confusing. The most often used names are: hybrid, nanohybrid, composite or nanocomposite. In our opinion, these materials are hybrids because they meet the definition of the hybrid material (have new properties/functions which component materials did not possess, or interpenetrated components are on a scale of less than 1 µm) [51]. Therefore, in the further part of this paper, we will call these materials hybrids. The main advantage of rGO hybrids is the elimination of the disadvantages of the particular components. Generally, rGO hybrids are characterized by: (i) low working temperature, (ii) high electrical conductivity and (iii) huge active sites.

In this paper, we present the most recent advances in rGO hybrid materials developed for NO_2_ detection. We discuss the properties of these materials and, especially, the mechanism of their interaction with NO_2_. Understanding the processes taking place on the surface of rGO sensors exposed to NO_2_ in air is a key issue in developing sensitive and thermodynamically stable sensor layers. In this paper, we also present current problems occurring in this field. In our considerations, we will focus on resistance sensors, in which the resistance varies with the presence of an analyte (NO_2_). A simplified principle of operation of such a sensor is presented in Section 3.

## 3. Principle of Operation of a Semiconductor Resistance Sensor

**Figure 4:**
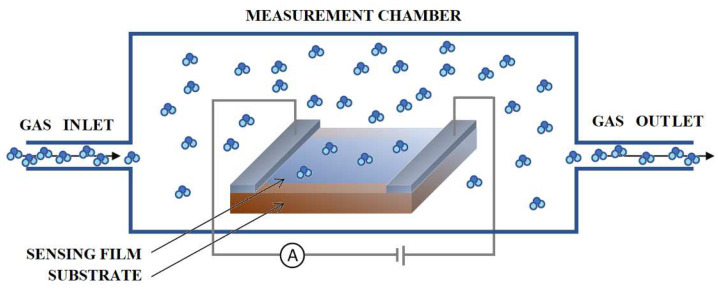
The simplified principle of operation of a n-type semiconductor resistance sensor.

The simplified principle of operation of a n-type semiconductor resistance sensor is shown in Figure 4.

**Figure 5:**
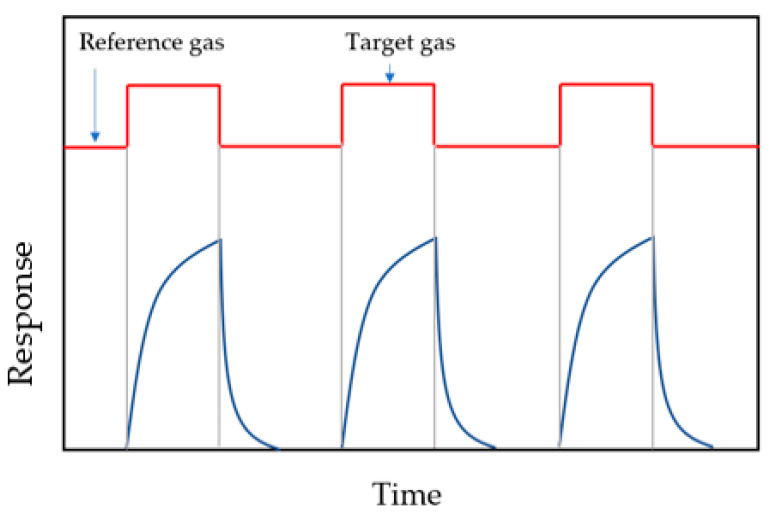
Illustration of the response signal of a semiconductor resistance sensor to the target gas.

A voltage is applied to the sensor which causes the current flow in the electric circuit. When gas appears, the gas molecules interact with the semiconductor surface to form chemical or Van der Walls bonds between the gas molecules and the adsorbent material. This process is called chemisorption and physisorption, respectively. Depending on the type of gas (reducing or oxidizing), electrons can be transferred from the surface of the sensor to the adsorbed gas molecules or in the opposite direction. This causes a change in the concentration of electric charge in the subsurface layer. The change in the concentration of electric charge in this area causes a change in the bands bending near the surface of the semiconductor. As a result, the resistance of the sensitive layer changes. The change in the semiconductor resistance causes a change in the electric current. By measuring the electric current (I) in the electric circuit and knowing the voltage across the sensor $\left(V\right)$, the sensor resistance can be easily determined using the Ohm’s equation: R=V/I. Since the absolute change in resistance does not say much about the sensitivity of the sensor, the relative change in resistance is determined using one of the following equations: $S=\frac{R_{g}-R_{r}}{R_{r}}$, $S=\frac{R_{r}}{R_{g}}$, $S=\frac{R_{g}}{R_{r}}$, where $R_{r}$ and $R_{g}$ are the sensor resistance in reference to atmosphere and in investigated gas, respectively. The higher the sensitivity of the sensor layer, the higher the parameter S. If the test gas and the reference gas are dosed sequentially in the measuring chamber, periodic peaks will be visible on the diagram of sensor signals over time, as shown in Figure 5. Since the operating temperature of a gas sensor determines its sensitivity, the sensor is heated to the temperature where its sensitivity is the highest. 

## 4. Mechanism of NO_2_ Detection in rGO Hybrid Materials

The most often reported rGO hybrid materials for NO_2_ detection are: rGO/SnO_2_, rGO/SnS_2_, rGO/ZnO, rGO/ZnO/NiO, rGO/Fe_2_O_3_ and rGO/polypropyrolle/CuO_2_. We describe the properties of these materials in separate subsections. In each case, the sensing mechanism is identified and discussed.

### 4.1. Reduced Graphene Oxide and Tin (IV) Oxide (rGO/SnO_2_)

**Figure 6:**
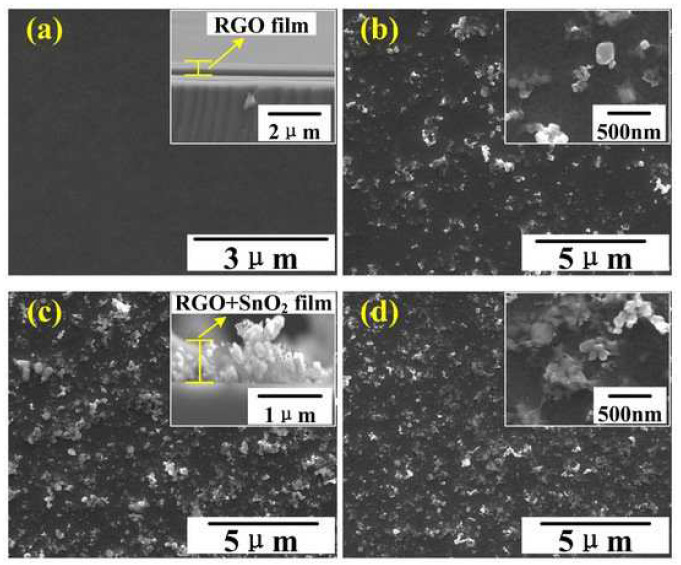
SEM images of: rGO (**a**), 9.7% rGO/SnO_2_ (**b**), 5.1% rGO/SnO_2_ (**c**) and 2.6% rGO/SnO_2_ (**d**). Reprinted from Ref. [54]. The mixture of rGO and SnO_2_ was prepared using the air brush method.

Tin (IV) oxide (SnO_2_) is a n-type semiconductor [5,50,52] with a good physical and chemical stability [53]. It belongs to non-toxic [53] and inexpensive material [53]. It has low surface area [50], high working temperature [50,53] and low electrical conductivity at room temperature [52]. In contact with NO_2_, its conductivity decreases [53]. The combination of rGO nanoflakes with micro- and nanoparticles of SnO_2_ causes a reduction in SnO_2_ grains and limitation of its aggregation [53]. SEM images of rGO and rGO/SnO_2_ with various concentrations of rGO are shown in Figure 6.

The surface area of rGO/SnO_2_ is much greater than both materials separately [50] and contains a high density of interface states. Its resistance is smaller than pure SnO_2_ [50,53]. In the aspect of NO_2_ detection, rGO/SnO_2_ exhibits enhanced response level, shorter recovery time and long-term stability [50]. Its sensitivity strongly depends on the rGO concentration [50,55] and, generally, increases with increasing rGO concentration [50]. The rGO concentration also determines the conductivity type of the rGO/SnO_2_ hybrid [56]. The improved properties of the rGO/SnO_2_ hybrid are associated with the following reasons [50,56]:

1. reduced graphene oxide has a favored porosity and broad specific surface area that provides more active sites for NO_2_ adsorption;
2. high carrier mobility of rGO allows it to increase an electron transport between NO_2_ molecules and conduction band of rGO/SnO_2_ hybrid;
3. p–n heterojunctions may enhance the NO_2_ adsorption due to more highly active sites, and it can cause a broader electron depletion layer.

**Figure 7:**
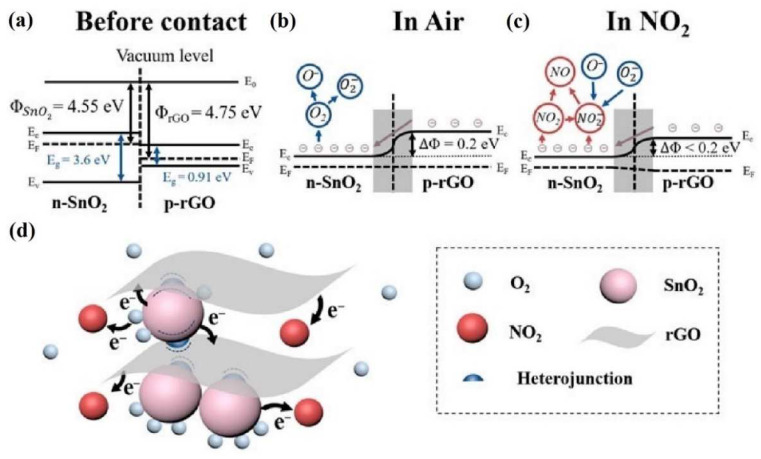
Band diagram of n-type SnO_2_ and p-type rGO before contact (**a**), rGO/SnO_2_ heterojunction in air (**b**) and in NO_2_ (**c**), possible NO_2_ gas-sensing mechanism of rGO/SnO_2_ (**d**). Reprinted from Ref. [57].

The schematic band diagram of SnO_2_ and rGO before contact, rGO/SnO_2_ heterojunction in air and in NO_2_, as well as the mechanism of rGO/SnO_2_ interaction with NO_2_ are shown in Figure 7.

The introduction of rGO to SnO_2_ causes a formation of the p–n heterojunction. Due to the difference in Fermi levels in both materials, electrons flow from SnO_2_ to rGO. This causes a formation of the depletion layer at the interface of both materials (there is an increase in electron concentration in the rGO and an increase in hole concentration in the SnO_2_). When rGO/SnO_2_ is exposed to air, oxygen is absorbed from the air on the rGO/SnO_2_ surface (Equation (1)) [56].

(1) $$O_{2}\left(\mathrm{gas})\;\to \;O_{2}(\mathrm{ads}\right)$$

Next, electrons are transferred from the conduction band of the rGO/SnO_2_ to oxygen resulting in the formation of negative oxygen ions according to the following reactions [56]:

(2) $$O_{2}\left(\mathrm{ads})+e^{-}\;\to \;O_{2}^{-}(\mathrm{ads}\right)$$

(3) $$O_{2}^{-}\left(\mathrm{ads})+e^{-}\;\to \;2O^{-}(\mathrm{ads}\right)$$

(4) $$O^{-}\left(\mathrm{ads})+e^{-}\;\to \;O^{2-}(\mathrm{ads}\right)$$

This causes an increase in the width of the depletion layer and, as a result, the increase in structure resistance. When NO_2_ appears, it is adsorbed on the rGO/SnO_2_ surface (Equation (5)).

(5) $${\mathrm{NO}}_{2}\left(\mathrm{gas})\;\to \;{\mathrm{NO}}_{2}(\mathrm{ads}\right)$$

Next, it reacts with both the negative oxygen ions $O^{-}$ and electrons from the layer, according to the following reactions:

(6) $${\mathrm{NO}}_{2}(\mathrm{ads})+O^{-}(\mathrm{ads})+2e^{-}\;\to \;{\mathrm{NO}}_{2}^{-}\left(\mathrm{ads})+O^{2-}(\mathrm{ads}\right)$$

(7) $${\mathrm{NO}}_{2}(\mathrm{ads})+O^{-}(\mathrm{ads})+2e^{-}\;\to \;{\mathrm{NO}}_{2}^{-}\left(\mathrm{ads})+O^{2-}(\mathrm{ads}\right)$$

This process leads to a further increase in the depletion layer and increase in the sensor’s resistance.

### 4.2. Reduced Graphene Oxide and Tin (IV) Sulfide (rGO/SnS_2_)

**Figure 8:**
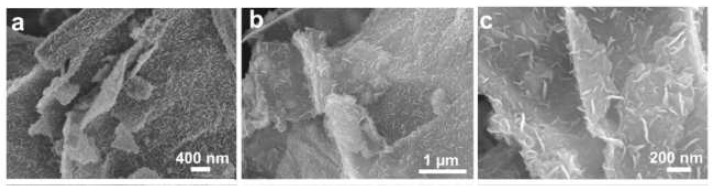
FESEM image of: rGO/Sn (**a**), rGO/SnS2 with different magnification (**b**) and (**c**), part of the figure. Reprinted from Ref. [58].

Tin (IV) sulfide (SnS_2_) is a n-type semiconductor [8] which exhibits poor sensor properties for NO_2_ detection at room temperature [49]. At this temperature, its resistance is too high to be measured [8,49]. Although it decreases with increasing working temperature, the probability of its oxidation to SnO_2_ increases, as well. The combination of rGO nanoflakes with SnS_2_ microstructure (Figure 8) decreases the sensor working temperature. For example, for undoped SnS_2_, the working temperature equals 200 °C while for 1.9% rGO/SnS_2_, it drops to 25 °C.

**Figure 9:**
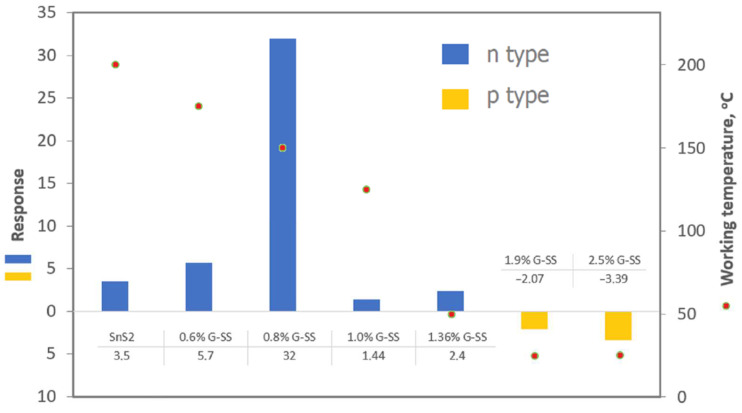
Comparison of sensing response and working temperature among SnS_2_ and G-SS (rGO/SnS_2_) [49].

However, an increase in rGO concentration has a negative influence on the sensitivity of the hybrid. For example, for a structure with concentrations of 0.8% rGO (Figure 9), a clear response is observed, while for a structure with concentrations of 1% of rGO, it decreases several dozen times. Increasing rGO concentration also causes deterioration of the response and recovery time and changes the type of hybrid conductivity from n to p [49].

The surface area of rGO/SnS_2_ is greater than both materials separately [49]. The combination of rGO with SnS_2_ causes the formation of C=C, C-O and C-S bonds. The presence of C-S bonds causes vacancies in the structure which facilitate the excitation of electrons into the conduction band. Furthermore, such vacancies are additional active centers for NO_2_ adsorption [49].

When SnS_2_ is combined with rGO, electrons are transferred from rGO to SnS_2_ and the depletion region is formed at the interface of both materials (there is an increase in electron concentration in the SnS_2_ and increase in hole concentration in the rGO) [59]. This creates an electrostatic field which, as a consequence, leads to a reduction in activation energy for adsorption/desorption of the target molecules. When the structure is exposed to NO_2_, electrons are ejected from rGO/SnS_2_. This leads to the reduction in the electron-depletion region in SnS_2_ and an increase in the hole-depletion region in the rGO.

### 4.3. Reduced Graphene Oxide and Zinc Oxide (rGO/ZnO)

**Figure 10:**
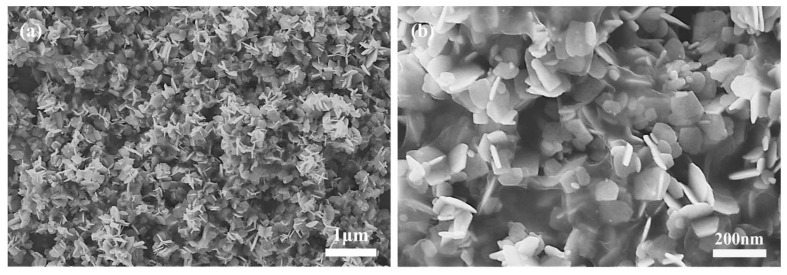
SEM images of rGO/ZnO_1-x_ composites: (**a**) low and (**b**) high magnification. Reprinted from Ref. [64].

Zinc oxide (ZnO) is a n-type semiconductor [46,60,61], which is characterized by good temperature and chemical stability [62,63]. It exhibits poor conductivity at room temperature, therefore, it is used at high temperature (150–450 °C) [60,62,64]. The combination of rGO nanoflakes with ZnO nanoparticles improved its conductivity, provided more active sites to absorb NO_2_ molecules and reduced working temperature to room temperature [46,62]. The changes in the rGO/ZnO resistance due to the NO_2_ are greater than that of pure ZnO [62,63]. The rGO/ZnO hybrid can be a n-type [62] or p-type [60] semiconductor, depending on the fabrication method. Authors [46] note that the combination of rGO and ZnO effectively prevents the agglomeration of ZnO and also prevents stacking between sheets, which increases the specific surface area. The SEM image of the rGO/ZnO hybrid is shown in Figure 10.

The sensor mechanism of rGO/ZnO interaction with NO_2_ is similar to the mechanism of pure ZnO [10,62]. In contact with air, oxygen is adsorbed on the surface of the receptor layer according to Equation (8).

(8) $$O_{2}\left(\mathrm{gas})\;\to \;O_{2}(\mathrm{ads}\right)$$

**Figure 11:**
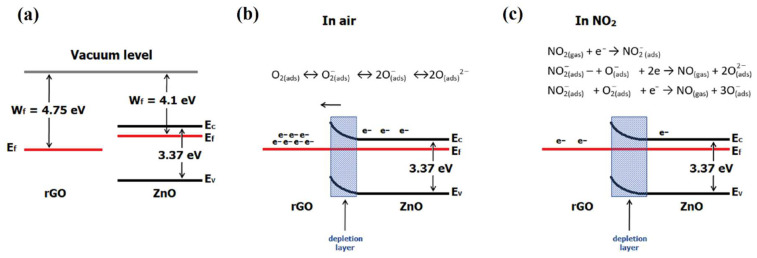
Band diagram of ZnO and rGO (**a**), possible mechanism of rGO/ZnO in air (**b**) and in NO_2_ (**c**).

Oxygen takes a free electron from ZnO and becomes an anion (Equation (2)). When NO_2_ appears, NO_2_ molecules are adsorbed on the rGO/ZnO layer according to reaction 5. The adsorbed NO_2_ reacts with a free electron from ZnO forming ${\mathrm{NO}}_{2}^{-}$ ions (Equation (7)). NO_2_ also reacts with the previously formed oxygen ions and with a free electron from ZnO, forming ${\mathrm{NO}}_{3}^{-}$ ions (Equation (9)). Both of these reactions consume free electrons, which causes a decrease in conductivity and, thus, an increase in resistance. Figure 11 shows the band diagram of ZnO and rGO (a) and the possible gas sensing mechanisms of rGO/ZnO nanocomposite in air (b) and in NO_2_ (c).

(9) $$2{\mathrm{NO}}_{2}(\mathrm{ads})+O_{2}^{-}+e^{-}\to \;2{\mathrm{NO}}_{3}^{-}$$

### 4.4. Reduced Graphene Oxide, Nickel Oxide and Zinc Oxide (rGO/NiO/ZnO)

**Figure 12:**
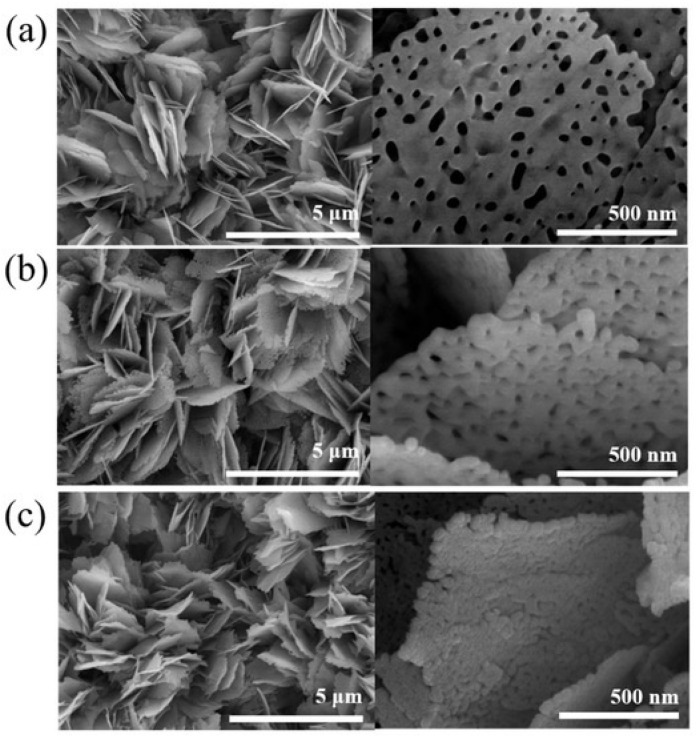
SEM images of pristine porous ZnO nanosheets (**a**) and porous ZnO/NiO composite nanosheets (**b**) and (**c**). Reprinted from Ref. [66].

In the literature, there are also attempts to combine rGO nanoflakes with several metal oxides nanocomposites. For example, the authors of [61] proposed a concept of combination of rGO with NiO and ZnO. Such a combination allows a layer to be obtained with higher sensitivity (defined as R_gas_/R_air_) than for pure ZnO (Figure 12a) and also for the NiO/ZnO composite (Figure 12b,c). As mentioned earlier, ZnO is a n-type semiconductor while rGO is a p-type semiconductor. Nickel oxide is a p-type semiconductor [61] which has high working temperature and poor selectivity [65]. Combining rGO with ZnO/NiO obtains a material similar to a n-type semiconductor. The mechanism of NO_2_ detection in rGO/ NiO/ZnO nanocomposites is as follows.

**Figure 13:**
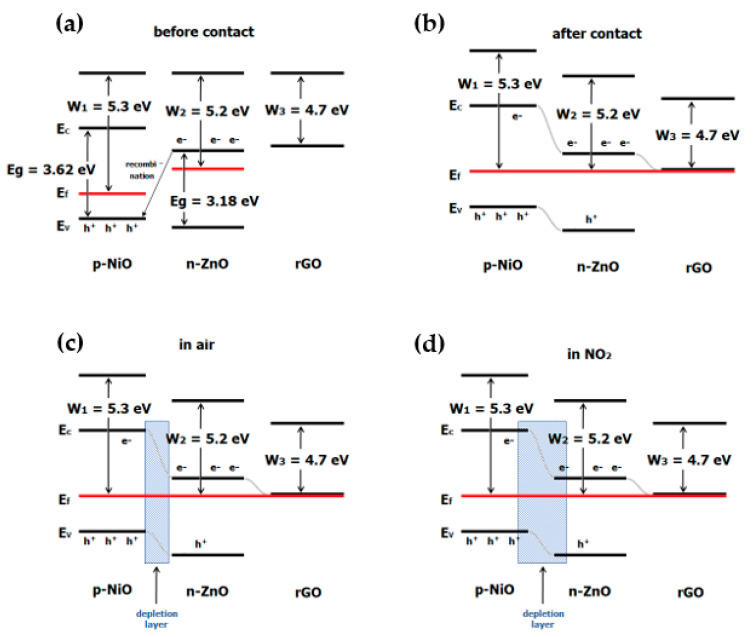
Band diagram of NiO/ZnO before (**a**) and after contact (**b**) and diagrams of gas adsorption on rGO/NiO/ZnO in air (**c**) and in NO_2_ (**d**).

When ZnO is combined with NiO, electrons flow from the ZnO layer to the NiO layer until the Fermi levels align. This creates an electron-depleted layer on the ZnO side and a hole-depleted layer on the NiO side (Figure 13). When the NiO/ZnO layer adsorbs nitrogen dioxide, it will donate electrons, causing deeper depletion on the ZnO side. At the same time, electrons from NiO will flow to NO_2_, which will increase the concentration of holes on the NiO side. As a result, an increase in the response is observed [61]. Reduced graphene oxide nanoflakes in the NiO/ZnO micro- and nanostructure is responsible for the acceleration of electron transfer. This is related to the velocity of adsorption/desorption of the gas and the reactions between the gas and the receptor material [61]. Moreover, rGO provides many additional active sites [61].

### 4.5. Reduced Graphene Oxide and Iron (III) Oxide (rGO/Fe_2_O_3_)

**Figure 14:**
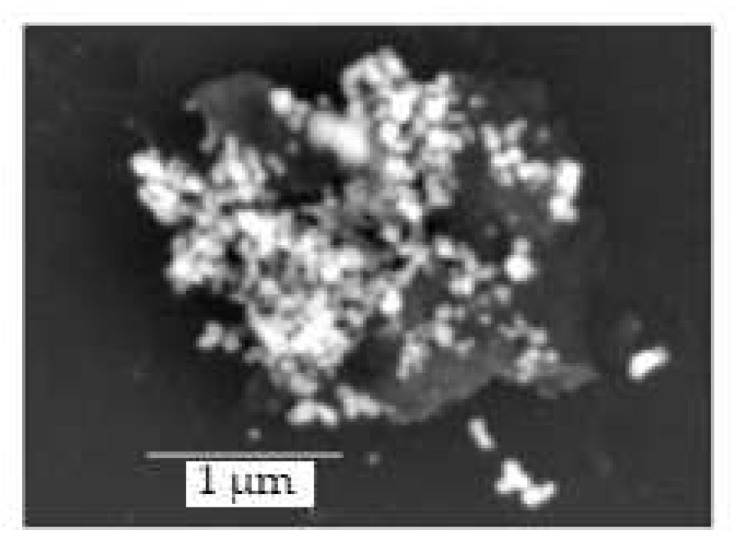
SEM image of GO/Fe_2_O_3_ structure. Reprinted from Ref. [67].

Iron (III) oxide (Fe_2_O_3_) is a n-type semiconductor [67] which is characterized by high electron mobility and chemical stability [68]. It exhibits poor sensitivity to NO_2_ detection at room temperatures. The combination of rGO nanoflakes and Fe_2_O_3_ nanoparticles allows a material similar to a p-type semiconductor to be obtained, which is more sensitive for NO_2_ than both materials separately [67]. The SEM image of GO/Fe_2_O_3_ is shown in Figure 14.

**Figure 15:**
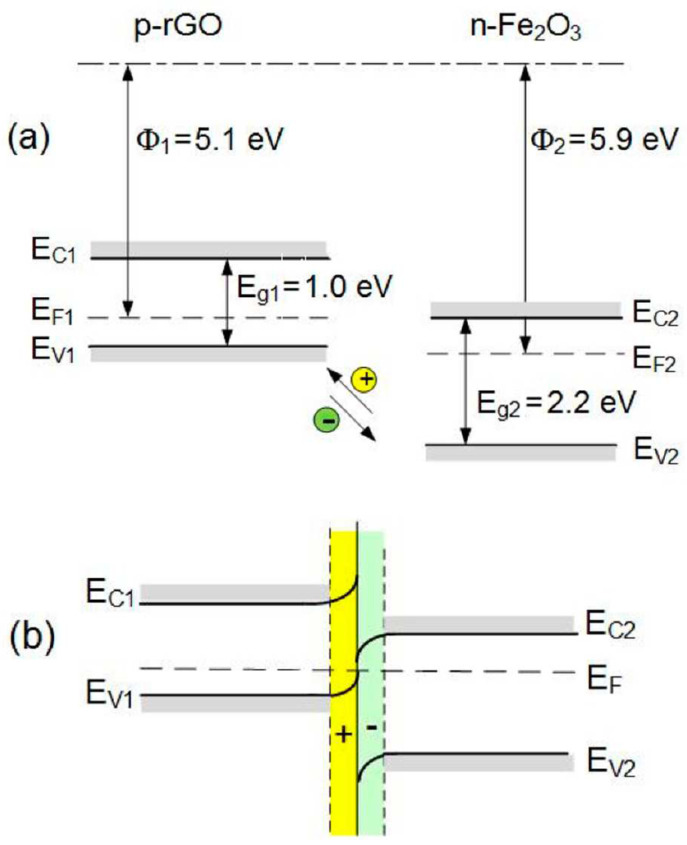
Band diagram of rGO and Fe_2_O_3_ before (**a**) and after contact (**b**). Reprinted from Ref. [67].

**Figure 16:**
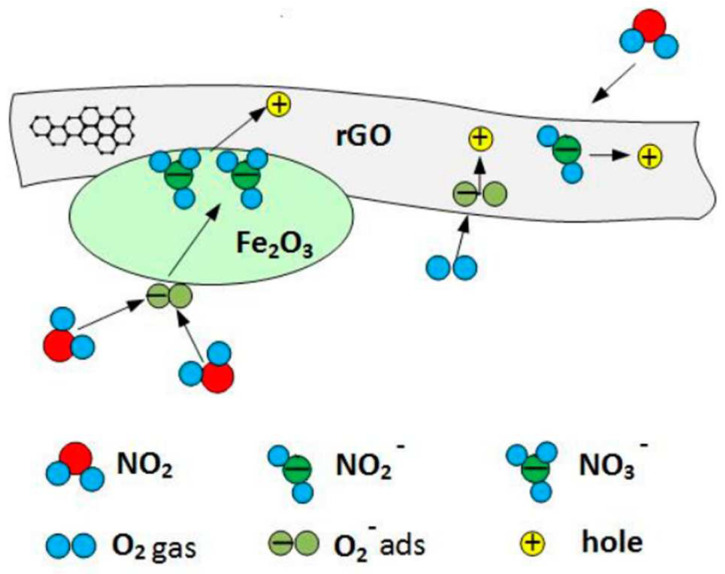
Possible NO_2_ gas-sensing mechanism of rGO/Fe_2_O_3_. Reprinted from Ref. [67].

Combination of Fe_2_O_3_ grains and rGO causes a formation of a heterojunction [67] (Figure 15) in which electrons are transferred from rGO to Fe_2_O_3_ [67]. rGo/Fe_2_O_3_ exhibits additional active sites (defects and/or oxygen functional groups) which improve the sensitivity [68]. Reduced graphene oxide, due to its excellent conductivity, can increase the conductivity of the structure [68]. When the structure is exposed in air, oxygen is adsorbed on the surface of rGO/Fe_2_O_3_ in the forms of $O_{2}^{-}$, $O^{2-}$ and $O^{-}$ ions [48]. These ions react with NO_2_ molecules in the surrounding atmosphere (10) [67] (Figure 16) and, as a result, an unbalanced negative charge is formed on the Fe_2_O_3_ surface. To neutralize this charge, electrons from the rGO are transferred. This generates holes in the graphene oxide [67] and, finally, causes a reduction in the structure resistance [67].

(10) $$2{\mathrm{NO}}_{2}(\mathrm{gas})+O_{2}^{-}\mathrm{ads}+e^{-}\to \;2{\mathrm{NO}}_{3}^{-}(\mathrm{ads})$$

### 4.6. Reduced Graphene Oxide, Polypyrrole and Copper (I) Oxide (rGO/PPy/Cu_2_O)

**Figure 17:**
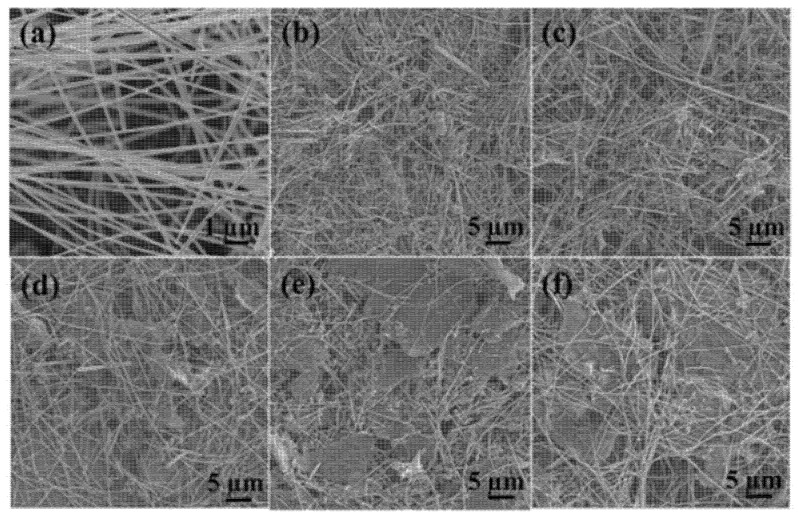
SEM images of PPy (polypyrrole)/Cu_2_O nanowires hydrothermally prepared at 120 °C (**a**), rGO/PPy/Cu_2_O after high-temperature reduction with mass ratio: 0.08 (**b**), 0.1 (**c**), 0.12 (**d**), 0.15 (**e**) and 0.2 (**f**). Reprinted from Ref. [69].

Polypyrrole (Figure 17a) is a p-type semiconductor characterized by high chemical stability, high conductivity, redox reversibility, simple preparation and low cost [69]. Therefore, polypyrrole nanostructures are a promising candidate for sensor application. Polypyrrole in combination with rGO (Figure 17b–f) prevents the accumulation/fusion of graphene sheets thanks to electrostatic repulsion between polypyrrole nanoparticles [69]. Additionally, it can enlarge the specific surface area, increase active sites and enhance the adsorption capacity of rGO, improving the affinity for gas [69].

Copper (I) oxide (Cu_2_O) is also a p-type semiconductor which is characterized by low cost, ease of synthesis and stability [70]. Cu_2_O have been widely studied for sensing applications in papers [69,71,72,73,74,75,76]. In combination with rGO, Cu_2_O, such as polypyrrole, can enlarge the specific surface area, increase active sites and enhance the adsorption capacity of rGO, improving the affinity for gas [69]. Moreover, Cu_2_O can prevent the restacking of graphene sheets and overcome inferior gas selectivity of graphene. 

In the case of NO_2_ detection, the combination of rGO/PPy/Cu_2_O exhibits better NO_2_ sensing performance than PPy/Cu_2_O composite [69]. When rGO/Cu_2_O/PPy composite is exposed in air, oxygen is adsorbed on its surface in the form of $O_{2}^{-}$ ions:

(11) $$O_{2}(\mathrm{gas})+e^{-}\;\to \;O_{2}^{-}(\mathrm{ads})$$

After the introduction of nitrogen dioxide, the NO_2_ molecules are trapping electrons from the hybrid material according to reaction (12) and from the oxygen ions (reaction (13)):

(12) $${\mathrm{NO}}_{2}(\mathrm{gas})+e^{-}\;\to \;{\mathrm{NO}}_{2}^{-}(\mathrm{ads})$$

(13) $${\mathrm{NO}}_{2}(\mathrm{gas})+O_{2}^{-}+2e^{-}\;\to \;{\mathrm{NO}}_{2}^{-}(\mathrm{ads})+2O^{-}$$

**Figure 18:**
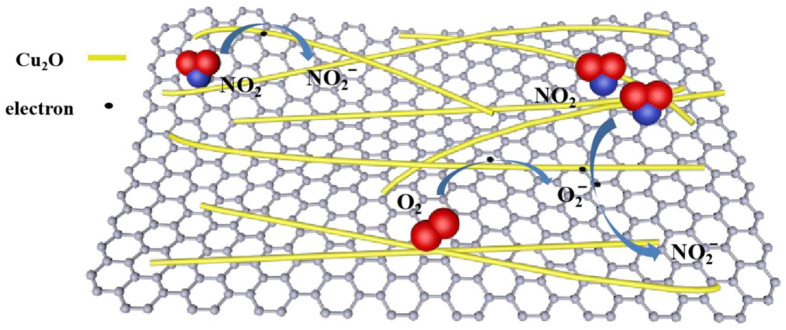
Possible NO_2_ gas-sensing mechanism of rGO/PPy/Cu_2_O. Reprinted from Ref. [44].

As a consequence, the concentration of holes increases in the hybrid material [69]. The high hole mobility of polypyrrole and graphene flakes favors carrier transport and migration to the electrode. Rapidly reducing the electron concentration and increasing the hole concentration reduces the sensor resistance. The schematic diagram of the sensing mechanism of rGO/PPy(polypyrrole)/Cu_2_O is shown in Figure 18.

In Table 1, we summarize and compare the NO_2_ sensing performance of the discussed rGO-based hybrids.

## 5. Remarks on WBS–rGO Hybrid Sensor Structures

The qualitative analysis of the influence of the reduced graphene oxide on the sensor properties of the discussed semiconductors (in particular, oxide semiconductors), in terms of their interaction with NO_2_ in air must take into account the fact that they were technologically obtained in the form of nanostructures with a highly developed surface.

Inorganic semiconductor nanostructures (WBS) with a wide band gap (ΔEg > 2 eV), at room temperature, have a relatively low concentration of free electrons in the conduction band. They are characterized by slow reaction kinetics: semiconductor–gas analyte (NO_2_). Therefore, the amount of electrons available for the effective charge transfer between the adsorbed gas molecules and the surface of the active layer is usually insufficient for its observation in the form of electrical signal. Generally, there are several ways to increase the concentration of electric charge in the conduction band, such as increasing temperature, reducing the bandgap of the semiconductor by introducing defects and impurities, lighting, etc. These kinds of problems and effects were discussed in detail in [10]. In particular, an increase in temperature above 100 °C not only increases the carrier density in the conduction band, but also reduces the effect of humidity on the sensitivity of detected gases (as water molecules adsorbed on the receptor material are heated and evaporated). The increase in temperature improves the sensor’s operation. The maximum response is usually at about 200 °C. Above this temperature, the response decreases (resistance increases) due to the sharp increase in the desorption rate of the analyte from the sensor surface. Heating also increases the energy consumption of the gas sensor.

The physical and chemical properties of these semiconductor nanostructures are largely determined by the specific surface area, electronic properties of this surface and the density of interface states in the bandgap of semiconductor.

Electrons from the conduction band, located in the interface states, create an unbalanced electric charge which increases the depletion region. For this reason, the sensitivity of such nanostructures on the oxidizing gases (including NO_2_) at low (room) temperatures is low. The interactions of NO_2_ with semiconductor structures are relatively weak and exhibit mainly a chemical nature, hardly reversible. The so-called “detoxification process” by treating the structure with air or nitrogen (NO_2_-free), at room temperatures, is very slow (it can take tens or event hundreds of minutes). To reduce this time, the structure temperature is increased to 400 K or even more. However, usually, after these processes, the electrical resistance of the structure does not return to its initial value.

The rGO micro- and nanoflakes are characterized by p-type electrical conductivity. In the previously discussed works, the base sensor material was the semiconductor, while the rGO flakes served as conducting channels between the WBS semiconductor structures, enabling more efficient transport of electric charge. The presence of unsaturated bonds on the rGO surface also creates active adsorption centers. Thus, compared to “pure” semiconductors (without rGO flakes) in the WBS + rGO structures, additional active sites are available for anchoring gaseous molecules. The WBS + rGO heterostructure is a random network of semiconductor nanostructures bridged by rGO nanoflakes. The difference of the Fermi levels of the n-type WBS and the p-type rGO may lead to the formation of p–n microjunctions at the phase boundaries. Before exposure to gas, the resistance for both “pure” semiconductor sensors and WBS + rGO heterostructures is mainly influenced by the adsorption of oxygen from the air. The presence of rGO increases the surface area for the adsorption of chemisorbed oxygen and target gas. Usually, the oxygen molecules from the air are adsorbed on the semiconductor surface and at the grain boundaries in the forms of $O_{2}^{-}$, $O^{-}$ or $O^{2-}$ ions. Next, these ions capture free electric charge from the conduction band of semiconductors that creates a charged area on the sensor surface. The mechanism of NO_2_ detection by structure based on a “pure” semiconductor (without rGO flakes) mainly consists in modifying the charged region formed by oxygen after exposure to the gas.

For WBS+rGO heterostructures, the depletion area formed at the inter-grain boundaries between the n-type semiconductor and the p-type rGO also affects the electrical resistance of the sensor structure. The increase in sensor resistance observed after exposure to a gas analyte is due to a change in the concentration of charge carriers in the conduction band, caused by gas molecules adsorbed on the sensor surface. When the sensor is exposed to NO_2_ (which is electrophilic in nature), the incident gas molecules capture free electrons directly from the conduction band of the structure and are adsorbed on the surface. Nitrogen present in the NO_2_ molecule has one unpaired electron, which promotes the chemisorption of NO_2_ molecules at the surface. The large surface offered by semiconductor nanostructures additionally supports the increased adsorption of gas molecules. Since the electron affinity of NO_2_ (2.27 eV) is higher than oxygen (0.45 eV), the absorbed NO_2_ molecules interact with the adsorbed oxygen ions and capture their electrons. The flow of electric charge from the surface of the WBS + rGO sensor to the molecules of the detected gas and from the adsorbed oxygen additionally increases the width of the depletion region, which leads to a significant increase in the sensor resistance.

## 6. Conclusions

Adding rGO to n-type oxide semiconductors, whose surface is negatively charged by electrons from the volume of the semiconductor and by electrons from the surrounding atmosphere located in the interface states with high density, weakens the hybridization of this surface.

Due to the presence of rGO in the semiconductor nanostructures, the interaction of NO_2_ with the surface of the semiconductor exhibits more physical nature than chemical, as is observed for the “pure semiconductor”. This new situation also causes the interaction of NO_2_ with the surface of the semiconductor to be more selective (rGO molecules both limit the amount of negative charge accumulated in the surface states and reduce the intensity of the electric field generated by the electric charge in the interface states). It is also important that rGO molecules form relatively large nanostructures that affect the distribution of the electric field in the area where they are located.

In the case of semiconductor nanostructures obtained by hydrothermal methods, both the shapes of the nanostructures and their sizes and electrical properties (their resistivity) depend on the technology of their production. In the sensor aspect, the optimal rGO concentration for different semiconductors, and for different production technologies, is different. There cannot be too few rGO nanostructures, because they will not sufficiently modify the surface of semiconductor nanostructures (their surface electrical properties). There cannot be too many rGO nanostructures because they will “dominate” the ongoing interactions of semiconductor nanostructures with NO_2_; first of all, they will “separate” NO_2_ and oxygen molecules too much from semiconductor nanostructures.

The physical-type interaction between semiconductor nanoparticles and NO_2_ particles occurs to a greater extent in WBS+rGO structures than in WBS without rGO. This causes semiconductor nanostructures containing rGO to be much easier to detoxify. The process of detoxification does not require elevated temperature and is significantly faster—it takes several or several dozen seconds. The analyses carried out in this paper indicate that the modification of the physicochemical properties of the nanostructures of semiconductors, by the use of rGO nanoflakes, can significantly improve the sensor properties of such structures.

## Figures and Tables

**Table 1:** Summary of rGO-based hybrids used for NO_2_ detection and their performance characteristics.

Hybrid	Temperature (°C)	Concentration of NO_2_ (ppm)	Sensitivity	Equation of Sensitivity	Reaction/ Regeneration Time (s)	Ref.
rGO/SnO_2_	55	100	1.079	$S=R_{\mathrm{air}}/R_{\mathrm{gas}}$	-/373	[53]
rGO/SnS_2_	80	11.9	56.8%	$S=100\%\cdot \frac{R_{\mathrm{gas}}-R_{\mathrm{air}}}{R_{\mathrm{air}}}$	360/3180	[59]
rGO/ZnO	RT	1	2.07	$S=R_{\mathrm{gas}}/R_{\mathrm{air}}$	150/55	[62]
rGO/NiO/ZnO	140	2	80.1	$S=R_{\mathrm{gas}}/R_{\mathrm{air}}$	39/16	[66]
rGO/Fe_2_O_3_	RT	5	3.86	$S=R_{\mathrm{air}}/R_{\mathrm{gas}}$	76/946	[48]
rGO/PPy/Cu_2_O	25	50	42.5%	$S=100\%\cdot \frac{R_{\mathrm{gas}}-R_{\mathrm{air}}}{R_{\mathrm{air}}}$	-/~200 ^1^	[69]

^1^ under auxiliary irradiations of an ultraviolet lamp.

## Data Availability

Not applicable.
